# Evaluating Social Media as a source of Mental Health Information: Insights from Mental Health Practitioners

**DOI:** 10.1192/j.eurpsy.2025.596

**Published:** 2025-08-26

**Authors:** J. X. Chua, A. Alasiam, K. L. C. Ng

**Affiliations:** 1 Department of Emergency and Crisis Care, Institute of Mental Health, Singapore, Singapore

## Abstract

**Introduction:**

Social media has significantly changed the way we communicate, interact, and access mental health information for both the public and practitioners. Research indicates that rising rates of suicidal behaviors among adolescents may be linked to increased screen time on social media (Balt et al., 2023). Excessive use of online social networks can exacerbate self-harm and suicidal thoughts in vulnerable young people (Memon et al., 2018). Given Emergency Mental Health Professionals are most often in contact with suicidal patients, we developed a survey to gain a deeper understanding of their practices and attitudes toward social media platforms.

**Objectives:**

We aim to explore social media usage patterns among mental health practitioners in Singapore’s sole psychiatric emergency department, assess their views on the impact of social media as a mental health resource, and evaluate whether they believe suicide posts on social media should be treated with the same seriousness as traditional suicide notes.

**Methods:**

The authors surveyed 58 mental health professionals - psychiatrists, nurses, psychologists, social workers, and pharmacists - who worked during 2023-2024. The survey, consisting of 19 multiple-choice questions, assessed attitudes toward mental health-related internet technologies and the perceived advantages and disadvantages of social media, based on a previous scientific paper titled “The Role of Social Media as a Resource for Mental Health Care”. Responses were measured on a Likert scale (1 = Strongly Disagree to 5 = Strongly Agree). SPSS 16.0 was used to analyze correlations between demographics and attitudes toward social media as a mental health resource.

**Results:**

Of the practitioners, 58.6% viewed social media’s impact on mental health as negative. Notably, 32.4% of those with less than one year of experience held this view, compared to just 8.8% of those with 6 to 9 years of experience. A significant negative correlation was found between actively following mental health content on social media and the belief that social media increases suicide risk among vulnerable individuals (P = 0.003, R = -0.389). However, years of work experience did not significantly correlate with this belief (P = 0.213).

**Conclusions:**

The study finds that while mental health professionals generally view social media negatively, those who engage with mental health content online are less likely to associate it with increased suicide risk. Our limited literature review found no similar studies, and we aim to provide new insights into how familiarity with mental health content influences professional attitudes. Expanding the research beyond Emergency Department practitioners could reveal how demographic factors shape opinions on social media.

**Disclosure of Interest:**

None Declared

